# ASPHER Statement: Déjà vu? Planning for the Covid-19 Third Wave and Planning for the Winter 2021–22

**DOI:** 10.3389/ijph.2021.1604361

**Published:** 2021-08-23

**Authors:** Vasco Ricoca Peixoto, John Reid, Henrique Lopes, Vladimir Prikazsky, Ines Siepmann, Jose M. Martin-Moreno, John Middleton, on behalf of ASPHER COVID task force

**Affiliations:** ^1^ NOVA National School of Public Health, Public Health Research Centre, Universidade NOVA de Lisboa, Lisbon, Portugal; ^2^ Comprehensive Health Research Centre, Universidade Nova de Lisboa, Lisbon, Portugal; ^3^ Department of Public Health and Wellbeing, Chester University, Chester, United Kingdom; ^4^ Unit of Public Health, Healthcare Sciences Institute, Universidade Católica, Lisbon, Portugal; ^5^ Independent Public Health Expert, Prague, Czechia; ^6^ ASPHER Young Professional, Brussels, Belgium; ^7^ Medical School and INCLIVA-Clinical Hospital, University of Valencia, Valencia, Spain; ^8^ Department of Public Health, Wolverhampton University, Wolverhampton, United Kingdom

**Keywords:** Public Health, COVID-19, pandemic, third wave, winter planning

## Introduction

A “third wave” of the pandemic has arrived in Europe. The Delta variant of SARS-COV2, is now widespread. Relaxations in pandemic control measures have been accompanied by surges in cases [1]. Concerns we reported ahead of the second wave are still relevant today, conveying a sense of *deja-vu* on last year’s concerns and recommendations [2]. Scientific knowledge is incomplete and we cannot yet appreciate the potential impact of SARS-COV2 on human and animal health, and on social and environmental conditions. How countries collectively respond will determine which road the pandemic takes, and how well we can control the pandemic in Europe and globally [3].

## ASPHER Believes Countries Must

### 1 Address the Uncontrolled Spread of Infections

We are not achieving population “herd” immunity at current levels of vaccine coverage. It is reckless to assume we can open up society and remain shielded from the worst effects of the virus. Allowing further uncontrolled spread of the virus creates opportunities for further variants of concern to emerge, at least one of which will be vaccine resistant [3].

Priority must be given to expanding genetic sequencing to detect virus mutations, with internationally agreed standards and shared resources and support [3].

Continued social distancing measures and wearing of face masks is necessary irrespective of vaccination status [4]. Improving the effectiveness of contact tracing and financial support for self-isolation is needed [5].

Countries should critically review their policies with regard to mass gatherings, learning from previous experience of superspreading events [6] and evaluation of the experience of the Euros 2020.

EU institutions should agree a strong common European policy on border controls, harmonized and effectively implemented by each Member State [3].

### 2 Recognise the Increasing Range of Clinical Presentations

There is widespread under-ascertainment of cases of COVID-19 infection and a high frequency of asymptomatic infection [3]. Testing for COVID-19 needs be extended to a broader range of suspicious symptoms. The public needs to recognize the widening range of symptoms of COVID-19 and why they should be tested, even if their symptoms are mild [3].

### 3 Recognise and Address the Widening Range of Syndromes and Clinical Consequences of “Long COVID”, “Persistent COVID” and COVID Related Medical Conditions

Health-care services must prepare to respond to COVID-19 associated medical conditions [3]. Enhanced surveillance is needed for COVID-19 sequelae and COVID-associated medical conditions. There should be an international agreement on categories of long COVID manifestations and a surveillance system implemented [3].

### 4 Rethink Inadequate Testing Tools and Strategies and Improve Public Understanding of the Limits of Tests

The systematic replacement of PCR tests by rapid tests has increased the risk of false negatives making it difficult to control transmission. Governments need to see testing as part of the toolbox for reducing transmission, but recognise its limitations and convey accurate information the general public [3].

### 5 Continue to Implement Mass Vaccination and Consistent Equitable Coverage of Their Population

Countries must improve vaccination coverage rapidly. They must strengthen efforts to address vaccine hesitancy [7]. Continued vigilance in surveillance of vaccine efficacy is needed. Countries must ensure that vaccines are distributed equitably in their communities, and internationally [8].

### 6 Address Unmet Health Care Needs

Countries still need to address unrecognised, untreated or uncontrolled long-term conditions and conditions requiring surgery during the pandemic. Health services must take advantage where there is a decrease in COVID-19 cases requiring hospitalisation to address the backlog in regular care. Governments must resist relaxations which risk an increase in COVID-19 hospital bed-occupancy and prevent any recovery in routine and emergency care [2].

### 7 Plan for Winter

In addition to planning for COVID-19, countries must plan for a major flu outbreak this year. Flu was greatly suppressed during 2020, through high flu vaccination rates, social distancing and mask wearing. These will have impacted on transmission of any respiratory viruses, and are still needed in 2021 [9].

Enhanced Influenza, pneumococcal vaccine programmes and vitamin D supplementation also need to be implemented [2].

Countries should prepare for a cold winter [2], and for extreme and unpredictable weather events, like those happening with flash floods in Europe and with the extreme heat dome over the North Western Pacific region [10].

Efforts to stimulate economies by reducing societal COVID-19 restrictions will fail if the virus is not suppressed to very low levels [11]. Countries must protect the health and the social welfare of all their people [2, 3]. Countries must protect their children’s future; keeping schools open should be the priority [12, 13].

### 8 Appreciate Their Global Responsibility to all Other Nations of the World

We will not be free of the pandemic until we are all free of it [14]. It is grossly irresponsible for any country to abandon all public health and social protection measures. It is an action, not confined to their own borders. It will have wide-ranging impact across the globe and weaken global efforts to suppress the virus [15]. The possibility of perpetual COVID remains real [16].

### 9 Earn the Trust of Your People, Govern by Informed Consent and Support Communities and Individuals

Communication strategies must change from the message of protecting the health system capacity and the lives of older individuals, to protecting young individual’s health over the long term due to the risk of long COVID-19 sequelae [3]. Some of the new SARS-COV2 variants will have an impact on the effectiveness of vaccines and put in jeopardy the huge vaccination efforts that have been made. This will have consequences for public trust and vaccine confidence.

Communication of the medium-term risks is essential for people to understand and give their support preventive efforts. Our governments and public health experts must earn the trust of the public we serve, and consent to the measures which must be implemented [2, 3].

Our messages must be clear, consistent and unequivocal. We can be optimistic, but we cannot be complacent in our efforts towards the end of this pandemic.

## Society note

ASPHER is responsible and liable for the content. The statement was approved by an Editor-in-Chief but not externally peer reviewed.
